# Cortical diurnal rhythms remain intact with microglial depletion

**DOI:** 10.1038/s41598-021-04079-w

**Published:** 2022-01-07

**Authors:** Rocio A. Barahona, Samuel Morabito, Vivek Swarup, Kim N. Green

**Affiliations:** 1 Department of Neurobiology and Behavior, University of California, 3208 Biological Sciences III, Irvine, CA 92697 USA; 2 Mathematical, Computational and Systems Biology (MCSB) Program, University of California, Irvine, CA USA

**Keywords:** Circadian rhythms and sleep, Glial biology

## Abstract

Microglia are subject to change in tandem with the endogenously generated biological oscillations known as our circadian rhythm. Studies have shown microglia harbor an intrinsic molecular clock which regulates diurnal changes in morphology and influences inflammatory responses. In the adult brain, microglia play an important role in the regulation of condensed extracellular matrix structures called perineuronal nets (PNNs), and it has been suggested that PNNs are also regulated in a circadian and diurnal manner. We sought to determine whether microglia mediate the diurnal regulation of PNNs via CSF1R inhibitor dependent microglial depletion in C57BL/6J mice, and how the absence of microglia might affect cortical diurnal gene expression rhythms. While we observe diurnal differences in microglial morphology, where microglia are most ramified at the onset of the dark phase, we do not find diurnal differences in PNN intensity. However, PNN intensity increases across many brain regions in the absence of microglia, supporting a role for microglia in the regulation of PNNs. Here, we also show that cortical diurnal gene expression rhythms are intact, with no cycling gene changes without microglia. These findings demonstrate a role for microglia in the maintenance of PNNs, but not in the maintenance of diurnal rhythms.

## Introduction

While microglia are most known for immunosurveillance of the central nervous system, they are increasingly being studied in the context of their non-immunological roles. Microglia actively maintain tissue homeostasis throughout adulthood by clearing cellular debris via phagocytosis as well as remodeling synapses^1,2^. Recently yet another homeostatic function of microglia has been identified: the regulation of perineuronal nets (PNNs). PNNs are specialized extracellular matrix structures which surround the soma and proximal synapses of neuronal subsets. In the mammalian brain, these lattice-like structures are found across various regions such as the hippocampus, but most notably enwrap parvalbumin-expressing GABAergic interneurons in the cortex^3^. The plant lectin *Wisteria floribunda agglutinin* (WFA) is a histological marker widely used to visualize PNNs as it binds its key structural components: chondroitin sulfate proteoglycans (CSPGs). WFA specifically binds terminal N-acetylgalactosamine residues of chondroitin sulfate chains^4^ which surround the core protein. PNNs can also be detected by antibodies against the core protein component of the CSPG aggrecan (ACAN)^5,6^ which is required for the formation of PNNs^7^. Functionally, PNNs play an important role in experience-dependent neuronal circuit plasticity, as their formation during development coincides with the closure of critical periods^8,9^. Throughout adulthood, many studies support the notion that PNNs help consolidate and maintain memories over time^10,11^. PNNs are also involved in regulating neuronal firing activity^12^ as well as protecting neurons from oxidative stress^13^.

We have consistently observed that elimination of microglia via colony stimulation factor 1 receptor (CSF1R) inhibitors^14^ dramatically increases PNN coverage throughout the brain^15–18^. Furthermore, upon cessation of treatment with the CSF1R inhibitor PLX5622, microglia repopulate the brain and PNNs decrease back to basal levels^19^. Notably, elimination of microglia not only increases PNN coverage, but also improves mouse performance in spatial memory tasks^20^ highlighting a potential role for microglia-mediated PNN maintenance in memory consolidation as subsequent studies identified the importance of microglia in “forgetting” and long-term memory^21,22^. It has been reported that PNNs are regulated in a circadian and diurnal manner with dramatic remodeling occurring during sleep in both mice and rats^23,24^. A daytime decrease in PNN intensity followed by a nighttime increase was shown in regions associated with decision making, learning, memory processing, and sleep (prelimbic area (PLA) and infralimbic area (ILA) of prefrontal cortex (PFC), hippocampus, amygdala, thalamic reticular nucleus), in adult C57BL/6J mice.

Likewise, microglia show diurnal changes in form and function. Morphologically, murine cortical and hippocampal microglia have a ramified shape during the light-phase (sleep) and a hyper-ramified shape during the dark-phase (wakefulness) characterized by longer processes and increased branch points^25–27^. Functionally, microglia exhibit diurnal differences in immune activation—with peak cytokine gene expression during the light phase^28^—and selective phagocytosis of opsonized synapses during each sleep phase^29^. Cell-autonomous diurnal rhythms of clock gene expression also exist in BV-2 cells, a murine-derived microglial cell line^30^. Following traumatic brain injury, complement factor C1q which is primarily produced by microglia, mediates sleep spindle loss^31^. Cathepsin-S (*Ctss*), a microglial expressed gene whose product can degrade PNNs, has also been reported to fluctuate in a circadian and diurnal fashion^24,25,32^. Furthermore, it is reported that daytime decrease in PNNs is antiphase to the expression of *Ctss*^24^.

Microglial ablation via administration of diphtheria toxin in Cx3cr1-Dtr rats has also been reported to disrupt circadian rhythms^33^, suggesting that microglia themselves may be critical for circadian regulation. As such, we hypothesized that microglia may be (1) responsible for PNN remodeling during sleep, via the secretion of CTSS and other proteases, allowing for the consolidation of memory and (2) involved in maintaining circadian rhythmicity in the brain. Here, we set out to investigate the role of microglia in the regulation of PNNs, specifically whether microglia regulate PNNs in a diurnal manner. While we find differences in microglia morphology between the light-phase and dark-phase, we find no differences in PNN integrated density across a 24-h day. However, upon microglial depletion, we find that PNN integrated density increases in a region-specific manner. Interestingly, the absence of microglia does not disrupt diurnal gene expression patterns in the cortex, showing microglia do not play a critical role in the regulation of cortical diurnal rhythms.

## Results

**Figure 1:**
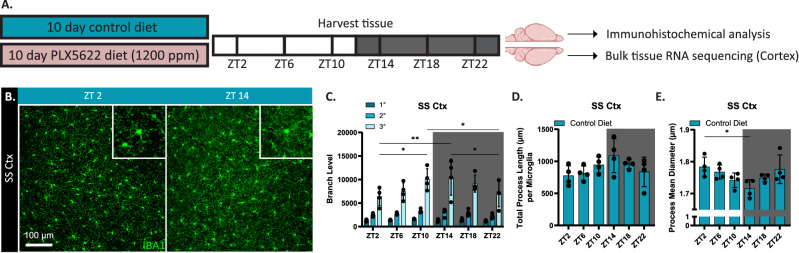
Diurnal differences in microglia morphology, but not perineuronal net intensity. (**A**) Schematic of experimental design. Immediately following a 10-day treatment with either PLX5622 or control diet, brains and peripheral tissues were harvested every 4 h for 24 h. (**B**) Representative 20× images of IBA1 (microglia) immunofluorescence in the SS Ctx from ~ 3-month-old wild-type (WT) mouse brains harvested at ZT2 and ZT14. Insets show microglia appear to be more ramified at ZT14 compared to ZT2. (**C**) Quantification of primary, secondary, and tertiary branching level from SS Ctx IBA1+ microglia showing an increase in tertiary branching during the light phase followed by a decrease during the dark phase. (**D**) No significant differences in total process length per microglia across a 24-h day were observed. (**E**) Process mean diameter is significantly smaller at ZT14 compared to ZT2. Statistical analysis used a two-way ANOVA with Tukey’s multiple comparisons correction for branch level; a one-way ANOVA with Tukey’s multiple comparisons correction for total process length (µm) per microglia and process mean diameter (µm) quantification. Significance indicated as *p < 0.05; **p < 0.01; ***p < 0.001.

Microglia are dependent on signaling through the CSF1R for their survival and we previously identified and optimized the specific CSF1R inhibitor PLX5622 in chow to produce rapid (within 3 days) and sustained microglial depletion^34^. To explore the role of microglia in regulating PNNs in a diurnal fashion, we fed 2.5-month-old male mice either control chow or chow containing the CSF1R inhibitor PLX5622 (1200 ppm) for 10 days to eliminate > 90% of microglia. Following treatment, brains and peripheral tissue were harvested at Zeitgeber times (ZT): ZT2, ZT6, ZT10, ZT14, ZT18, and ZT22 where ZT0 denotes the onset of the light-phase (daytime) and ZT12 the onset of the dark-phase (nighttime). One brain hemisphere was processed for immunohistochemical analysis and the other hemisphere microdissected for bulk-tissue RNA sequencing (RNAseq) of the cortex (Fig. 1A).

### Diurnal differences in microglia morphology

To assess whether microglia morphology differed between the light and dark phase, we employed immunohistochemistry to visualize IBA1+ microglia from brains harvested at different ZTs. With other studies reporting diurnal differences in cortical microglia^25,26^, we focused our morphological analysis on microglia in the somatosensory cortex (SS Ctx). We found cortical microglia appear morphologically different shortly after the onset of the light-phase and shortly after the dark-phase (Fig. 1B). With regards to degree of branching we observed the highest degree of tertiary branching at ZT14, indicative of increased ramification, and the least ramified microglia at ZT2 (Fig. 1C). To further confirm microglia are more ramified at the onset of the dark phase, we looked at total process length per cell (Fig. 1D) and process mean diameter (Fig. 1E). While there are no significant differences in total process length per microglia across 24 h, it appears microglial process length increases by 40% from ZT2 to ZT14 and subsequently decreases from ZT14 to ZT22 in a wave-like pattern. Process mean diameter follows the opposite pattern, where microglial processes are significantly thinnest at ZT14 compared to ZT2. These findings suggest microglia display diurnal morphological differences where microglia are most ramified with thinner processes at the onset of the dark phase.

### Perineuronal nets increase in a region-dependent manner upon microglial depletion

**Figure 2:**
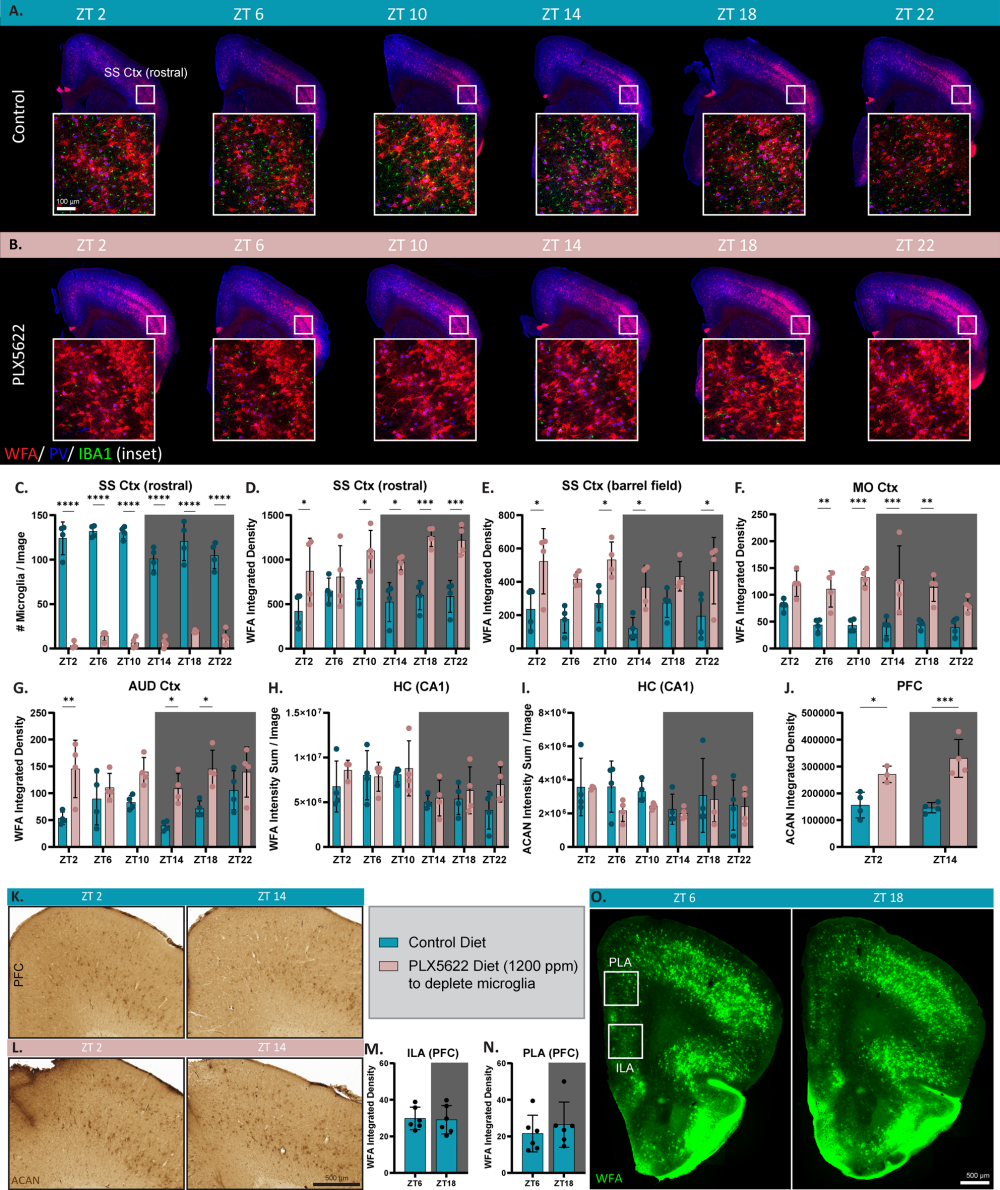
Perineuronal nets increase in a region-dependent manner upon microglial depletion. (**A**) Representative slide-scanned images of WFA (PNN) staining, parvalbumin, and IBA1 immunofluorescence reveal no diurnal changes in PNNs across 24 h and (**B**) a significant increase in PNNs with microglial depletion. (**C**) 10-day PLX5622 (1200 ppm) treatment is sufficient to eliminate >90% of microglia at all timepoints. (**D**–**G**) Quantification for WFA integrated density for the (**D**) rostral SS Ctx, (**E**) barrel field of the SS Ctx, (**F**) MO Ctx, and (**G**) AUD Ctx show no diurnal PNN differences in control mice and a significant increase in the PLX5622 treated mice. (**H**,**I**) Quantification of (**H**) WFA+ and (**I**) ACAN+ PNN intensity sum from 20× hippocampal CA1 confocal images show no diurnal differences in PNN intensity nor an increase in PNN intensity with microglial depletion. (**J**) Quantification of ACAN integrated density shows no difference between ZT2 and ZT14, but in the absence of microglia, ACAN integrated density significantly increases. (**K**) DAB staining with ACAN in WT control mice shows no drastic diurnal differences in PFC PNNs, but in (**L**) mice treated with PLX5622, ACAN+ PNN integrated density significantly increases. (**M**,**N**) Quantification of WFA integrated density in both the (**M**) infralimbic and (**N**) prelimbic area show no diurnal differences in PNNs between ZT6 (mid-light phase) and ZT18 (mid-dark phase). (**O**) Representative slide-scanned images of WFA (PNN) labeling in the PFC. Statistical analysis used a two-tailed unpaired t-test for ILA and PLA WFA Integrated Density quantifications and a two-way ANOVA with Tukey’s multiple comparisons correction the quantifications with PLX5622-treated groups. Significance indicated as *p < 0.05; **p < 0.01; ***p < 0.001.

Having observed diurnal differences in microglial morphology, we next sought to confirm that PNNs change in a diurnal manner and whether eliminating microglia would disrupt any changes. Brighter WFA staining is thought to be indicative of a more mature PNN composition^35^, therefore quantifying integrated density could provide insight into diurnal PNN remodeling. Counter to prior reports of diurnal PNN fluctuations, however, we find no diurnal changes in WFA+ PNN integrated density in SS Ctx (rostral and barrel field regions), motor cortex (MO Ctx), auditory cortex (AUD Ctx), hippocampus (CA1), ILA and PLA of the PFC (Fig. 2A,D–H,M–O). Staining against the PNN component ACAN also showed no diurnal differences in ACAN intensity in the hippocampus (CA1) and in ACAN integrated density in the PFC (Fig. 2I–K). Additionally, quantification of diffuse WFA+ PNN staining revealed no significant differences between ZT6 and ZT18 in the ILA, PLA, and SS Ctx (Fig. S3). We next confirmed effective depletion of microglia with PLX5622 treatment—quantification of IBA1+ microglia showed ~ 90% microglial loss (Fig. 2C). Examination of PNNs in the absence of microglia again show no diurnal changes but do show robust increases in WFA+ PNN integrated density in the SS Ctx (rostral and barrel field regions), MO Ctx, and AUD Ctx compared to microglia intact mice (Fig. 2B,D–G). Increases in ACAN+ PNN integrated density were also detected in the PFC (Fig. 2J,L) compared to control mice. In the CA1 region of the hippocampus, however, there are no increases in WFA+ PNN nor ACAN+ PNN intensity with microglial elimination (Fig. 2H,I).

### Cortical diurnal rhythms remain intact upon microglial depletion

**Figure 3:**
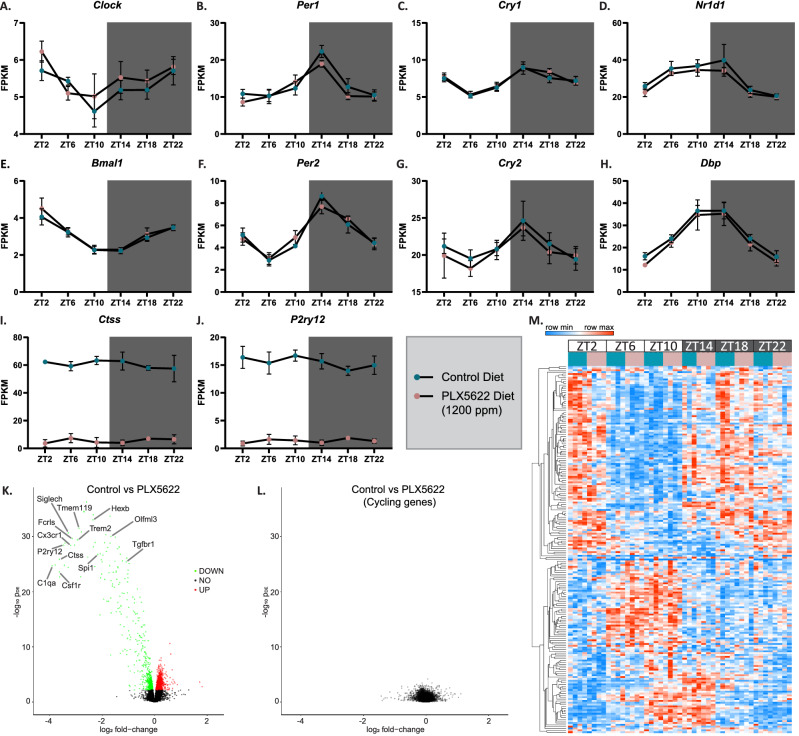
Cortical diurnal rhythms remain intact upon microglial depletion. (**A**–**H**) Cortical gene expression of canonical cycling genes (*Clock, Bmal1, Per1, Per2, Cry1, Cry2, Nr1d1, and Dbp*) across a 24-h day reveals no changes in rhythmicity in the absence of microglia (2 treatments, 6 groups per treatment, n = 4 per group). (**I**,**J**) Gene expression of (**I**) *Ctss* and (**J**) *P2ry12* does not fluctuate in a diurnal manner. (**K**) Volcano plot showing differentially expressed genes in the cortex of WT control mice compared to microglia-depleted mice (2 treatments, n = 24 per treatment). (**L**) Volcano plot showing no changes in cycling gene expression between WT control mice and microglia-depleted mice. (**M**) Heatmap of top 200 cycling genes shows diurnal expression patterns do not change upon microglial depletion.

To ensure that the lack of diurnal changes in PNNs was not due to an unexpected disruption of diurnal rhythms in our mice, we next looked at canonical “clock genes” via bulk-tissue RNA sequencing of cortical tissue. Importantly, circadian gene expression oscillations occur in the cortex^36^. Indeed, our data show that the control mice used in these experiments harbor intact diurnal rhythms shown by the oscillating expression of *Clock, Bmal1, Per1, Per2, Cry1, Cry2, Nr1d1,* and *Dbp* (Fig. 3A–H). With a recent study in rats reporting microglial ablation disrupts the circadian system and another study suggesting microglia contribute to the regulation of circadian amplitude in zebrafish^33,37^, we aimed to determine how eliminating microglia would affect diurnal rhythms in mice. Upon microglial elimination, we find no changes in diurnal clock gene expression compared to controls with intact microglia, indicating that microglia may not partake in the regulation of diurnal rhythms in the cortex. Furthermore, no significant changes in *Ctss* nor *P2ry12* expression were seen across the 24-h period, reflecting a lack of diurnal changes in these microglial expressed genes in the cortex (Fig. 3I,J; RNAseq data accessible at the Circadian Expression Browser website: http://swaruplab.bio.uci.edu:3838/circadian_shiny/). Differentially expressed genes were identified between control and PLX5622 treated groups (microglia-depleted) and shown as a volcano plot (Fig. 3K). As expected, gene expression changes reflect the loss of microglia from these brains. We further identified all cycling genes in control mice and found no significantly altered genes when compared to microglia-depleted mice (Fig. 3L). To illustrate this, the top 200 cycling genes are displayed as a heatmap and show no differences in rhythmic expression upon microglial elimination (Fig. 3M). Together, these data show that while some microglial functions may be regulated by diurnal rhythms, microglia themselves are not regulators of rhythmicity.

## Discussion

In confirmation of prior studies, we report that cortical microglia display diurnal morphological differences in the degree of ramification. Both the amount of tertiary branching and total process length per cell were highest at ZT14 and lowest at ZT2 while process mean diameter was lowest at ZT14 and highest at ZT2. These findings support work by others showing mean total length and number of microglial process branch points were significantly greater at ZT14 than ZT2^26^. However, while it has been reported that Ctss and P2ry12 are necessary for diurnal differences in morphology and that the expression of these genes oscillate in a diurnal manner, we did not find diurnal fluctuations in *P2ry12* nor *Ctss* expression, which suggests there may be other mechanisms contributing to diurnal morphological differences, at least in the cortex. With regards to *Ctss* expression across 24 h, it is important to note that existing data is contradictory, with some studies showing *Ctss* is upregulated during the light-phase and others showing it is upregulated during the dark-phase^24,32^. In a study where blood serum from healthy human subjects was collected over 24 h, no variations in CTSS concentration were found^38^, suggesting it is not regulated in a circadian manner in the periphery. Consistent with our data, in a study looking at sleep deprivation in 2-month-old C57BL/6J mice, RNAseq data from cortex of the undisturbed controls shows no changes in *Ctss* nor *P2ry12* expression between ZT0, ZT6, and ZT11 (timepoints beyond ZT11 were not investigated) where one would expect to observe a peak/ trough at ZT6 for rhythmic gene expression^39^.

Using a fluorescent IHC approach to investigate PNNs across six different ZTs, we found no evidence of diurnal changes in the intensity of these structures. To ensure that lack of diurnal differences in PNNs was not due to the method of detection, we utilized DAB staining to visualize the PNN component ACAN and found no diurnal differences in ACAN+ PNNs of the PFC. Our results run counter to previous findings, so a potential factor to consider for the lack of diurnal PNNs in this study include differences in vivarium housing conditions. While we also used adult male WT C57BL/6J mice, our mice were group-housed in standard cages rather than individually housed in wheel-running cages^24^. Additionally, since we investigated mice housed under light/dark conditions (diurnal) and not constant darkness conditions (circadian), it is possible that differences in PNNs or gene expression may have emerged under constant darkness conditions between control and microglia-depleted groups. Ultimately, through RNAseq we show mice used in this study had intact diurnal expression of the canonical cycling clock genes, and therefore felt confident proceeding with investigating diurnal endpoints. Upon microglial depletion with PLX5622, we found robust increases in PNN intensity in the PFC, SS Ctx, MO Ctx, and AUD Ctx, but not the CA1 region of the hippocampus, demonstrating that we had sufficient sensitivity to detect further increases in PNN intensity. These findings are supported by a study where microglial elimination with PLX3397 did not alter PNN intensity in the CA1 region^40^.

Importantly, this work is the first to show canonical cycling genes maintain their diurnal expression patterns in the absence of microglia, suggesting microglia may not be key regulators of rhythmicity in the brain. These findings are consistent with a recent study showing no overt variations in diurnal and circadian motor activity patterns between control and PLX5622-treated mice^41^. Counter to this, a previous study concluded that microglial ablation disrupts the circadian system in Cx3cr1-diphtheria toxin rats^33^. However, microglial ablation via diphtheria toxin conjures a cytokine storm^42^, which may have a larger role in the disruption of circadian rhythms rather than the depletion of microglia. Proinflammatory cytokines alter circadian gene expression in mouse cartilage and liver^43,44^, making it plausible that diphtheria toxin-induced inflammation may underlie circadian rhythm changes in the brain. Notably, disruption of core clock gene expression occurs 48 h after the first diphtheria toxin injection (corresponding to acute inflammation) and these differences resolve 56 h post injection (when microglia are still presumably absent, based on our experience with microglial repopulation dynamics)^33^. While the study in Cx3cr1-Dtr rats primarily focused on behavioral rhythmic changes, the study presented here explored rhythmic changes at the genetic expression level in the cortex of mice, therefore a direct comparison between the two studies cannot be made.

While CSF1R inhibition with PLX5622 is body-wide, depletion is restricted to microglia (and CNS macrophages) as they are uniquely dependent on CSF1R signaling for survival compared to the vast majority of peripheral macrophages^14,20^. Since our results are conclusively negative, with gene expression levels of cycling genes unchanged in the PLX5622-treated mice compared to control mice, we have no reason to believe that inhibition of CSF1R in peripheral myeloid-derived cells prevented the disruption rhythmic gene expression.

Altogether, while we find the expected diurnal changes in microglial morphology, and that microglial elimination consistently elevates PNN levels throughout the brain, we find no evidence of diurnal fluctuations of PNNs in untreated control mice. RNAseq from microdissected cortices show intact diurnal fluctuations in gene expression in both untreated and microglia-depleted mice, with no differential cycling genes identified. These results support the idea that PNNs may not be regulated in a diurnal fashion, and that microglia are not critical regulators of diurnal gene expression patterns in the adult cortex.

## Methods

### Animals

All animal experiments performed in this study were carried out in compliance with the ARRIVE guidelines, approved by the UC Irvine Institutional Animal Care and Use Committee (IACUC), and were compliant with ethical regulations for animal research and testing. Forty-eight 2-month-old adult male C57BL/6J mice were obtained from The Jackson Laboratory (JAX) and housed 4 to a cage in a 12:12 h light/dark cycle. Mice were allowed to acclimate to vivarium for a couple weeks before starting experiments. 24 mice were fed ad libitum control chow for 10 days, and the other 24 mice were fed PLX5622 (1200 ppm in AIN-76A chow) for 10 days to deplete microglia. Following the 10-day treatment with either control or PLX5622 chow, tissue from 4 male C57BL/6J mice was harvested every 4 h across the 24-h cycle at ZT2, ZT6, ZT10, ZT14, ZT18, and ZT22. A separate set of adult male mice, housed 3 per cage and fed control chow ad libitum, were used to ensure reproducibility of diurnal rhythms. Tissues from these mice were harvested at ZT6 and ZT18, 6 mice per time point. All mice were sacrificed via CO_2_ inhalation and perfused transcardially with ice-cold 1× PBS. Brains were extracted and dissected down the midline, with one half flash-frozen for subsequent RNAseq analyses, and the other half drop-fixed in 4% paraformaldehyde. Fixed brains were cryopreserved in 1× PBS + 0.05% NaN_3_ (sodium azide) + 30% sucrose at 4 °C, frozen and sectioned at 40 µm on a Leica SM2000 R sliding microtome and stored in a 1× PBS + 30% glycerol + 30% ethylene glycol solution at − 20 °C for subsequent IHC analyses.

### Immunohistochemistry

Primary antibodies used and dilutions are as follows: Iba1 (1:1000, 019–19741, Wako; and 1:500, ab5076, Abcam), *Wisteria floribunda agglutinin* (WFA) lectin (1:1000, B-1355, Vector Labs), Aggrecan (1:200, AB1031, Millipore), and Parvalbumin (1:500, MAB1572, Millipore).

As previously described^45^, sections were washed 3× 5 min in 1× PBS and immersed in normal serum blocking solution (5% normal serum + 0.2% Triton-X100 in 1× PBS) for 1 h. Tissue was then incubated overnight in primary antibody at the dilutions described above in normal serum blocking solution at 4 °C. The next day tissue sections were washed in 3× 5 min in 1× PBS before being placed in appropriate secondary antibody in normal serum blocking solution (1:200 for all species and wavelengths; Invitrogen) for 1 h. Tissue sections were then washed for 3× 5 min in 1× PBS before tissue was mounted and coverslipped. High resolution fluorescent images were obtained using a Leica TCS SPE-II confocal microscope and LAS-X software. To capture whole brain stitches, automated slide scanning was performed using a Zeiss AxioScan.Z1 equipped with a Colibri camera and Zen AxioScan 2.3 software.

### RNA sequencing

Whole transcriptome RNAseq libraries were produced from cortical tissue of control and PLX5622 treated mice sacrificed at ~ 3 months of age. RNA was isolated with an RNA Plus Universal Mini Kit (Qiagen, Valencia, USA) according to the manufacturer’s instructions. Library preparation, RNA sequencing, and read mapping analysis were performed by Novagene Co. Gene expression values for line graphs were normalized into FPKM (fragments per kilobase of transcript per million mapped reads). Heatmaps were created using Morpheus (Morpheus, https://software.broadinstitute.org/morpheus).

### Processing RNAseq data

The raw paired-end sequences from each sample were mapped to the mouse reference genome (GRCm38.p6) using STAR (v2.7.0f)^46^. Aligned reads were sorted and reads mapping outside of the 20 primary chromosomes were discarded from further analysis. Gene expression was quantified and normalized as transcripts per million mapped reads (TPM) using Salmon (v1.0.0)^47^ with the comprehensive gene annotation from Gencode. TPM data were assessed for effects from biological variables (treatment, group, RNA integrity number) and technical covariates related to sequencing quality, which were computed using PicardTools v2.1.1. The following linear model was used to account for these sources of variance in the expression matrix using R:

$${\text{lm}}\left( {{\text{Expression }}\sim {\text{ Timepoint }} + {\text{ Treatment }} + {\text{ Group }} + {\text{ Seq{-}}}{\text{PC1 }} + {\text{Seq{-}}}{\text{PC2 }} + {\text{ Seq{-}}}{\text{PC3}}} \right)$$

where Expression represents the TPM gene expression values; Timepoint, Treatment, and Group represent the relevant biological variables; and Seq-PC1, Seq-PC2, and Seq-PC3 sequencing metrics from PicardTools aggregated using principal component analysis (PCA). R-Shiny was used to create an interactive portal to explore the processed RNA-seq dataset (http://swaruplab.bio.uci.edu:3838/circadian_shiny/).

### Rhythmic gene expression analysis

The MetaCycle R package (v1.2.0) was used to identify rhythmic gene expression within the RNA-seq dataset^48^. The meta2d function in MetaCycle was used to perform JTK_Cycle analysis, an algorithm which identifies rhythmic genes in temporal expression data and estimates their amplitude, phase, and period length^49^. The limorhyde function (v 0.1.2) was used to perform fourier transformation on the time points for each sample in the expression dataset, and the first harmonic of this transformation was used to account for time in the subsequent differential expression tests^50^. Limma (v3.48.3) was used for differential gene expression analysis between Control and PLX5622 mice^51^. Differentially rhythmic genes and differentially expressed genes were computed using the limma functions lmFit followed by eBayes with the following design formulas respectively:

$${\text{dr}}\_{\text{design }} = {\text{ Expression }}\sim {\text{ Genotype }}* \, \left( {{\text{time}}\_{\text{cos }} + {\text{ time}}\_{\text{sin}}} \right)$$

$${\text{de}}\_{\text{design }} = {\text{ Expression }}\sim {\text{ Genotype }} + {\text{ time}}\_{\text{cos }} + {\text{ time}}\_{\text{sin}}$$

where expression is the normalized TPM expression values; Genotype denotes whether the sample was Control or PLX5622; time_cos and time_sin are based on the first harmonic of the previously described Fourier decomposition. Differentially rhythmic genes require a statistically significant interaction between the Genotype and the time components, while differentially expressed genes are solely dependent on the linear model coefficient for Genotype without additional interaction terms. Both differentially expressed and differentially rhythmic genes were corrected for multiple testing using the Benjamini–Hochberg procedure.

### Data analysis and statistics

GraphPad Prism (version 9) was used to perform statistical analysis. To compare two groups (same treatment, but different ZTs), the unpaired Student’s t-test was used. To compare six groups (same treatment, but different ZTs), a one-way analysis of variance (ANOVA) with Tukey’s multiple comparison correction was performed. To compare six groups (different ZTs) with different treatments (control versus PLX5622 diet), a two-way ANOVA with Tukey’s multiple comparison correction was used. For all analyses, statistical significance was accepted at p < 0.05. and significance expressed as follows: *p < 0.05, **p < 0.01, ***p < 0.001.

## Supplementary Information

Supplementary Figures.

## Data Availability

RNA sequencing data are available through GEO servers Accession GSE188989.

## Abbreviations

Acan: Aggrecan
AUD Ctx: Auditory cortex
Bmal1: Brain and muscle arnt-like protein 1
Clock: Circadian locomotor output cycles kaput
Cry: Cryptochrome
Ctss: Cathepsin-S
Iba1: Ionized calcium binding adaptor molecule 1
ILA: Infralimbic area
MO Ctx: Motor cortex
Per: Period
PFC: Prefrontal cortex
PLA: Prelimbic area
PNN: Perineuronal net
P2ry12: Purinergic receptor P2Y12
SS Ctx: Somatosensory cortex
WFA: *Wisteria floribunda agglutinin*
ZT: Zeitgeber time
