# Supplementary material for: Cortical diurnal rhythms remain intact with microglial depletion
Source: Sci Rep. 2022 Jan 7;12:114. doi: 10.1038/s41598-021-04079-w (PMC8742049; doi:10.1038/s41598-021-04079-w)
Supplement: Supplementary file 1 — Supplementary Figures. [file 41598_2021_4079_MOESM1_ESM.pdf]

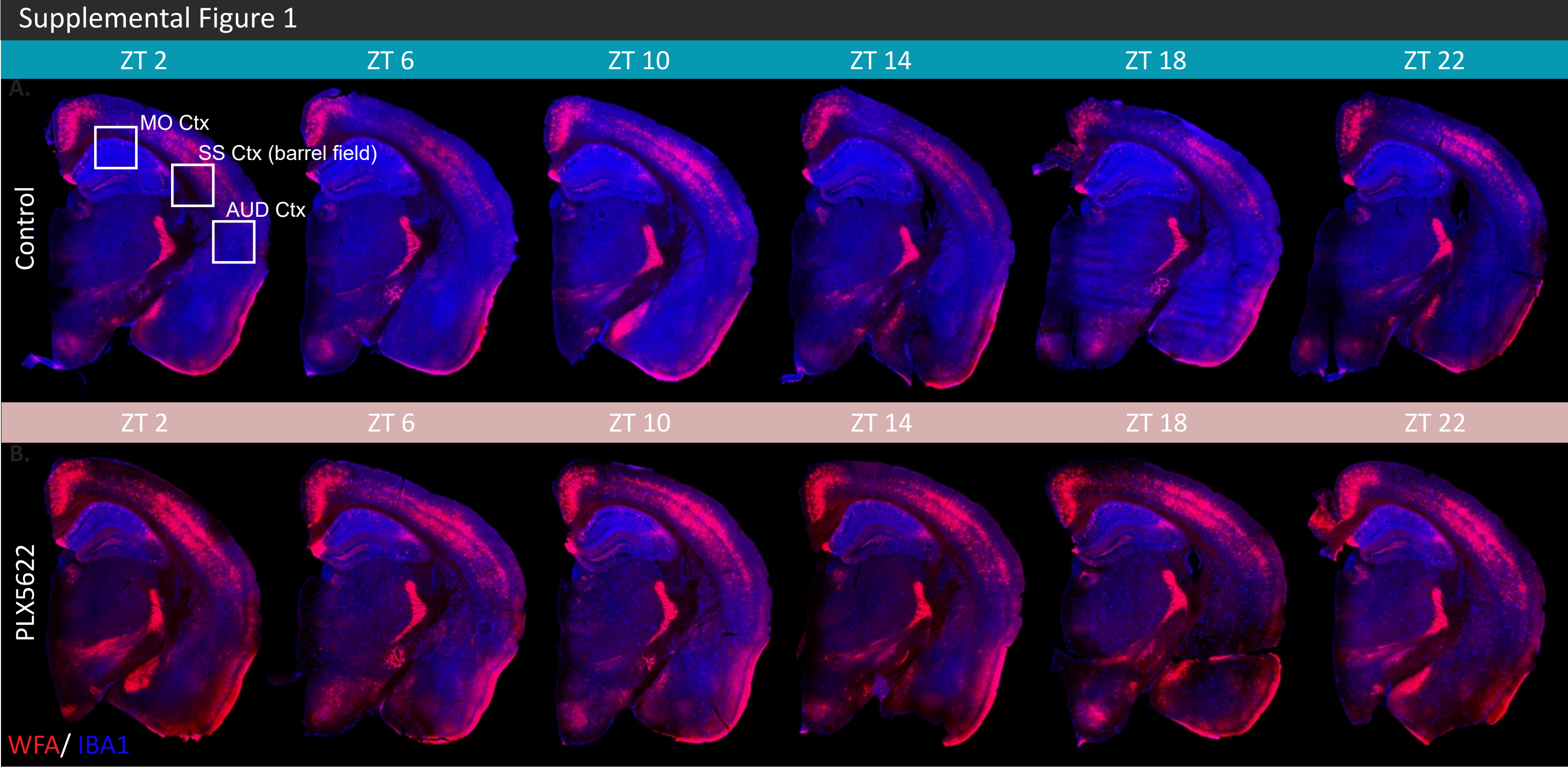

Figure S1 – Cortical perineuronal nets increase upon microglial depletion with PLX5622
(A) Representative slide-scanned images of WFA staining and IBA1 immunofluorescence in control mice and **(B)** PLX5622-treated mice corresponding to cortical quantifications in Figure 2.

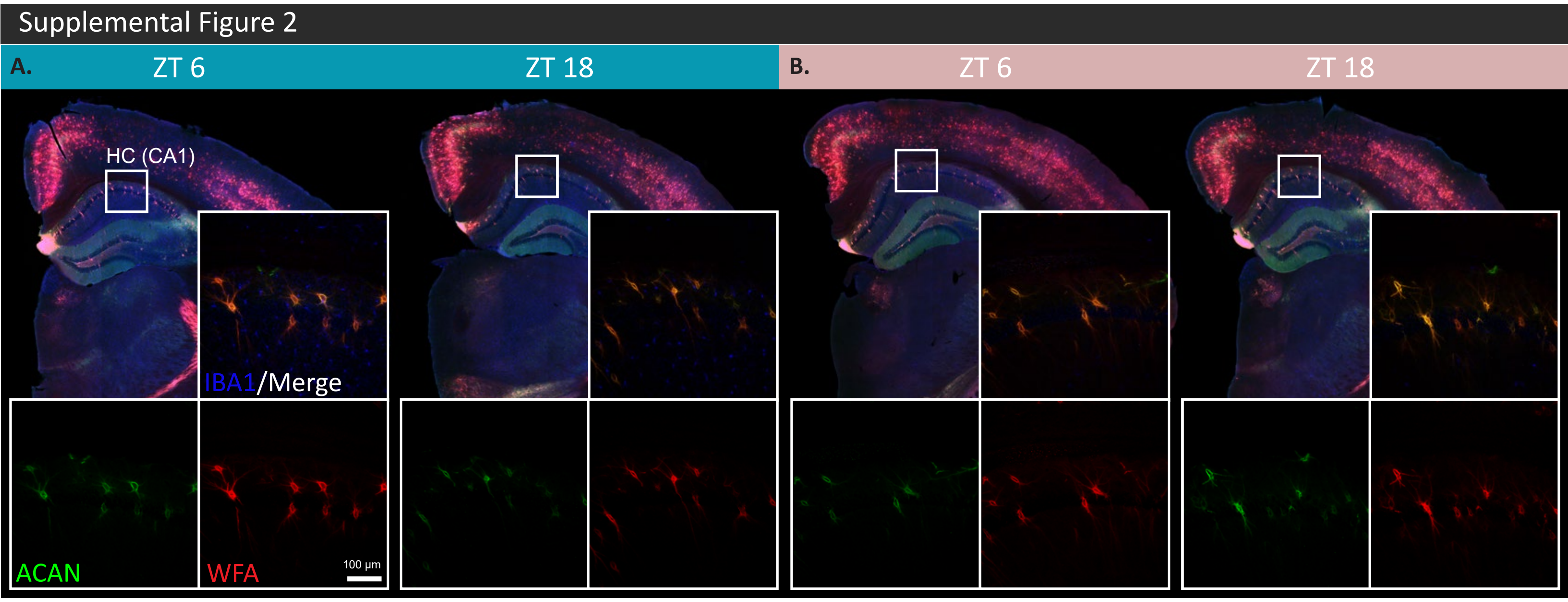

Figure S2 – Hippocampal perineuronal nets do not increase upon microglial depletion with PLX5622
(A) Representative slide-scanned and 20x confocal images of WFA staining, and ACAN and IBA1 immunofluorescence at ZT6 and ZT18 in control mice and **(B)** PLX5622-treated mice corresponding to hippocampal quantifications in Figure 2.

Supplemental Figure 3

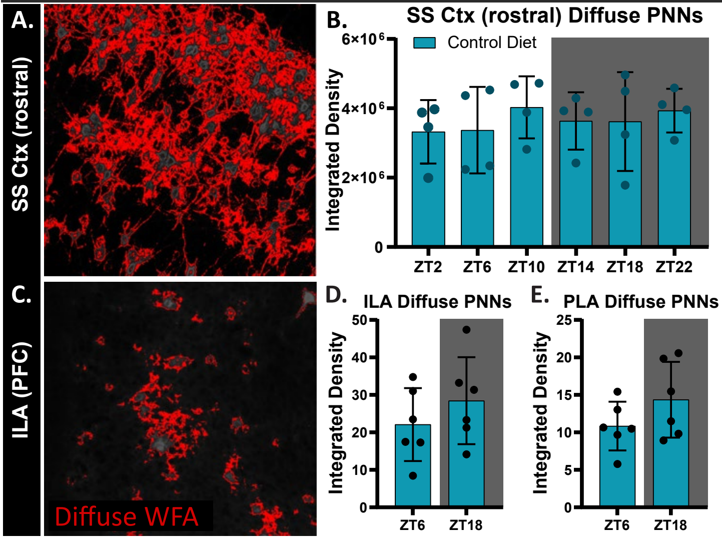

Figure S3 – No significant diurnal changes in diffuse PNNs in the cortex
(A) Image of SS Ctx PNNs showing WFA staining with diffuse compartment highlighted in red. **(B)** Quantification of SS Ctx diffuse PNN integrated density at ZT2, ZT6, ZT10, ZT14, ZT18, and ZT22. **(C)** Image of ILA PNNs showing WFA staining with diffuse compartment highlighted in red. **(D)** Quantification of ILA diffuse PNN integrated density at ZT6 and ZT18. **(E)** Quantification of PLA diffuse PNN integrated density at ZT6 and ZT18. Statistical analysis used a two-way ANOVA with Tukey's multiple comparisons correction for SS Ctx (rostral) diffuse PNNs and a two-tailed unpaired t-test for ILA and PLA diffuse PNN quantifications. Significance indicated as * $p<0.05$; ** $p<0.01$; *** $p<0.001$.
